# Comparison of lower extremity joint mechanics between healthy active young and middle age people in walking and running gait

**DOI:** 10.1038/s41598-019-41750-9

**Published:** 2019-04-03

**Authors:** Li Jin, Michael E. Hahn

**Affiliations:** 1 Neuromechanics Laboratory, University of Oregon, Eugene, OR USA; 2 Bowerman Sports Science Clinic, Department of Human Physiology, University of Oregon, Eugene, OR USA; 3 Present Address: Department of Physical Therapy & Rehabilitation Science, University of Iowa, Iowa City, IA USA

## Abstract

Progression of age can influence gait characteristics. Previous research has investigated lower extremity joint mechanics between young and elderly people in locomotion, however little is known about whether differences exist between young and middle age people. Ten young healthy subjects (22.8 ± 5.3 years) and ten middle age healthy subjects (50.7 ± 6.0 years) engaged in treadmill walking (from 0.8 to 2.0 m/s) and running (from 1.8 to 3.8 m/s). The middle age group had higher ankle plantar flexor moment angular impulse (p = 0.002), total support moment impulse (p = 0.016), and hip stance positive work (p = 0.029) across walking speeds. Additionally, the middle age group had higher knee flexion angle at ground contact in walking (p = 0.005) and running (p = 0.037). These findings indicate that moderate age affects changes in ankle and hip kinetic characteristics in walking, and knee kinematic patterns in both walking and running.

## Introduction

Human locomotion results from an integration of physiological and biomechanical factors^1^. These factors are affected by increasing age, and thus can influence changes in gait patterns. A decrease in preferred locomotion speed and step length have been reported as typical gait pattern changes associated with increased age^2–5^. This suggests that to achieve the same locomotion speed compared with young age people, there may be some compensatory mechanisms within the lower extremity system in middle-age and elderly individuals. Such compensatory mechanisms are likely associated with relevant gait kinematic and kinetic pattern changes.

Previous studies have investigated age effects on joint level kinematic and kinetic patterns in both walking^1,2,6–11^ and running^12,13^ gait. Older adults redistributed lower extremity joint moment and power to maintain similar gait performance compared with young people in walking^6^. Specifically, older adults tended to transfer the mechanical demands from distal to proximal in the lower extremity, by increasing net positive work at the hip to compensate for decreased work generated at the ankle^2,6,9^. In running conditions, elderly people tended to have less knee joint range of motion and a loss of shock-absorbing capacity compared with young people^12,13^. These observations indicate that the knee joint may become a “stiffer” system as age increases. While most of these studies focused on comparisons between young and older adults, little is known about whether there are differences in gait patterns and lower extremity joint mechanics across a smaller age range: between young and middle-age people in walking and running conditions. Moreover, previous comparisons were mainly focused on self-selected walking^2,7^ and running^12^ speeds. It remains unknown if there is an age effect on gait mechanics characteristics at the same locomotion task and speed condition. Specifically, this study sought to determine if there are differences in gait kinematic and kinetic characteristics between young and middle age adults, in a range of control speed conditions in both walking and running.

Joint torsional stiffness is a combination of joint level kinematic and kinetic variables in stance phase during locomotion. It reflects the sagittal plane dynamic loading response characteristics of a joint. Joint level stiffness has been reported to increase with age. This increase would be associated with relevant joint level kinetic changes, such as joint flexor/extensor moment and relevant stance phase extensor moment angular impulse, joint mechanical work, power absorption and generation^2^. The goal of the current study was to identify ankle, knee and hip joint stiffness patterns, joint level kinematic and kinetic characteristics between young and middle age groups while walking and running, across a range of speeds. A further purpose was to identify whether there is a compensatory mechanism among lower extremity joints in middle age people in a wide range of walking and running speeds. We hypothesized that the middle age group would have: (1) higher joint stiffness; (2) higher stance phase hip joint extensor moment angular impulse and positive work, lower ankle joint plantar flexor moment angular impulse and positive work; and (3) smaller joint angle range of motion compared with the young age group.

## Results

In both walking and running conditions, there was no significant difference between young and middle age subjects for the joint stiffness measures; *K*_*ankle*_, *K*_*knee*_ and *K*_*hip*_ (Table 1).

**Table 1:** Joint stiffness (Nm/kg/deg) between the young (n = 10) and middle age (n = 10) groups across walking and running speeds.

	Ankle		Knee		Hip	
	Young	Middle	Young	Middle	Young	Middle
***Walk***						
0.8 m/s	0.11 (0.09)	0.11 (0.05)	0.13 (0.05)	0.13 (0.07)	0.06 (0.03)	0.06 (0.07)
1.0 m/s	0.11 (0.07)^a^	0.16 (0.08)^a^	0.13 (0.04)	0.13 (0.05)	0.07 (0.03)	0.07 (0.02)
1.2 m/s	0.08 (0.04)^a^	0.11 (0.06)^a^	0.08 (0.01)	0.09 (0.02)	0.06 (0.01)	0.06 (0.02)
1.4 m/s	0.09 (0.03)	0.10 (0.06)	0.08 (0.02)	0.08 (0.02)	0.06 (0.01)	0.06 (0.02)
1.6 m/s	0.07 (0.02)^a^	0.13 (0.07)^a^	0.08 (0.02)	0.10 (0.04)	0.07 (0.02)	0.07 (0.03)
1.8 m/s	0.09 (0.02)^a^	0.12 (0.05)a	0.09 (0.01)	0.10 (0.03)	0.07 (0.01)	0.08 (0.03)
2.0 m/s	0.09 (0.02)^a^	0.13 (0.04)^a^	0.11 (0.02)	0.12 (0.04)	0.09 (0.02)	0.10 (0.05)
***Run***						
1.8 m/s	0.15 (0.05)^a^	0.22 (0.10)^a^	0.09 (0.02)	0.11 (0.03)	0.25 (0.11)	0.26 (0.17)
2.2 m/s	0.17 (0.05)	0.19 (0.04)	0.10 (0.03)	0.11 (0.02)	0.26 (0.15)	0.19 (0.04)
2.6 m/s	0.18 (0.08)	0.20 (0.04)	0.11 (0.03)	0.13 (0.03)	0.33 (0.14)	0.19 (0.04)
3.0 m/s	0.19 (0.05)	0.20 (0.11)	0.13 (0.03)	0.14 (0.04)	0.28 (0.06)	0.19 (0.05)
3.4 m/s	0.22 (0.09)	0.20 (0.05)	0.16 (0.05)	0.15 (0.06)	0.34 (0.08)	0.19 (0.08)
3.8 m/s	0.25 (0.07)^b^	0.21 (0.10)^b^	0.16 (0.05)	0.20 (0.09)	0.33 (0.10)	0.23 (0.07)

Sample Mean (SD). ^a^Large effect size between young and middle age group comparison (d > 0.5). ^b^Medium effect size between young and middle age group comparison (0.3 < d < 0.5).

In walking and running conditions, mechanical work at the joint (*W*_*joint*_) was generally similar between young and middle age groups for each joint (Tables 2 and 3). Swing phase *W*_*joint*_ data can be found in Supplementary Tables S2 and S3. However, during the stance phase of walking, ${W}_{hip}^{+}$ for the middle age group was higher than for the young age group (p = 0.029) across walking speeds from 1.0–1.8 m/s (Table 2). In the stance phase of walking, ${W}_{hip}^{-}$ for the middle age group was lower than for the young age group (p = 0.031) across walking speeds from 1.0–1.8 m/s (Table 2).

**Table 2:** Joint work (J/kg) between young (n = 10) and middle age (n = 10) groups in stance phase across walking speeds; Mean (SD). Note: Joint negative work data are in absolute values.

	Ankle		Knee		Hip^*^	
	Young	Middle	Young	Middle	Young	Middle
***Stance Phase Positive Work***						
0.8 m/s	0.13 (0.06)	0.12 (0.06)	0.10 (0.07)	0.05 (0.03)	0.09 (0.05)	0.08 (0.03)
1.0 m/s	0.18 (0.08)	0.18 (0.03)	0.10 (0.07)	0.10 (0.06)	**0**.**13 (0**.**05)**	**0**.**15 (0**.**04)**
1.2 m/s	0.18 (0.08)^a^	0.28 (0.18)^a^	0.11 (0.04)	0.14 (0.07)	**0**.**09 (0**.**04)**	**0**.**14 (0**.**07)**
1.4 m/s	0.25 (0.07)	0.23 (0.09)	0.13 (0.03)	0.11 (0.04)	**0**.**12 (0**.**03)**	**0**.**16 (0**.**06)**
1.6 m/s	0.27 (0.12)^a^	0.38 (0.18)^a^	0.18 (0.07)	0.23 (0.15)	**0**.**13 (0**.**05)**	**0**.**20 (0**.**08)**
1.8 m/s	0.31 (0.12)^a^	0.41 (0.20)^a^	0.22 (0.10)	0.21 (0.09)	**0**.**16 (0**.**07)**	**0**.**20 (0**.**06)**
2.0 m/s	0.34 (0.16)^a^	0.45 (0.17)^a^	0.22 (0.14)	0.22 (0.11)	**0**.**18 (0**.**08)**	**0**.**27 (0**.**08)**
***Stance Phase Negative Work***						
0.8 m/s	0.24 (0.07)	0.16 (0.05)	0.07 (0.06)	0.13 (0.08)	0.07 (0.06)	0.09 (0.07)
1.0 m/s	0.22 (0.05)	0.21 (0.05)	0.14 (0.14)	0.19 (0.12)	**0**.**11 (0**.**13)**	**0**.**10 (0**.**06)**
1.2 m/s	0.16 (0.09)	0.21 (0.11)	0.21 (0.18)	0.15 (0.03)	**0**.**23 (0**.**20)**	**0**.**10 (0**.**05)**
1.4 m/s	0.18 (0.05)	0.18 (0.04)	0.15 (0.03)	0.18 (0.10)	**0**.**13 (0**.**07)**	**0**.**12 (0**.**14)**
1.6 m/s	0.13 (0.05)	0.24 (0.14)	0.27 (0.24)	0.30 (0.24)	**0**.**32 (0**.**35)**	**0**.**16 (0**.**06)**
1.8 m/s	0.13 (0.05)	0.16 (0.10)	0.33 (0.26)	0.24 (0.11)	**0**.**36 (0**.**41)**	**0**.**17 (0**.**09)**
2.0 m/s	0.11 (0.03)	0.14 (0.16)	0.24 (0.08)	0.29 (0.17)	**0**.**22 (0**.**06)**	**0**.**16 (0**.**06)**

*Statistically significant difference between young and middle age groups across speeds are indicated in bold. ^a^Large effect size between young and middle age group comparison (d > 0.5).

**Table 3:** Joint work (J/kg) between young (n = 10) and middle age (n = 10) groups in stance phase across running speeds; Mean (SD).

	Ankle		Knee		Hip	
	Young	Middle	Young	Middle	Young	Middle
***Stance Phase Positive Work***						
1.8 m/s	0.46 (0.18)	0.49 (0.12)	0.20 (0.06)	0.23 (0.05)	0.04 (0.02)	0.05 (0.05)
2.2 m/s	0.51 (0.21)	0.49 (0.15)	0.23 (0.05)	0.25 (0.06)	0.05 (0.03)	0.09 (0.06)
2.6 m/s	0.46 (0.26)	0.56 (0.11)	0.24 (0.06)	0.25 (0.07)	0.13 (0.16)	0.10 (0.07)
3.0 m/s	0.50 (0.24)	0.52 (0.11)	0.30 (0.11)	0.25 (0.06)	0.14 (0.08)	0.16 (0.10)
3.4 m/s	0.52 (0.28)	0.55 (0.20)	0.29 (0.13)	0.29 (0.13)	0.21 (0.13)	0.19 (0.11)
3.8 m/s	0.70 (0.17)	0.62 (0.19)	0.30 (0.10)	0.23 (0.09)	0.21 (0.05)	0.24 (0.16)
***Stance Phase Negative Work***						
1.8 m/s	0.30 (0.09)	0.32 (0.06)	0.37 (0.17)	0.29 (0.09)	0.11 (0.07)	0.17 (0.06)
2.2 m/s	0.31 (0.10)	0.34 (0.08)	0.38 (0.13)	0.29 (0.13)	0.12 (0.07)	0.19 (0.08)
2.6 m/s	0.31 (0.09)	0.37 (0.09)	0.42 (0.22)	0.33 (0.12)	0.13 (0.11)	0.22 (0.11)
3.0 m/s	0.35 (0.14)	0.39 (0.12)	0.46 (0.19)	0.25 (0.13)	0.18 (0.11)	0.26 (0.13)
3.4 m/s	0.33 (0.14)	0.41 (0.19)	0.45 (0.14)	0.30 (0.14)	0.20 (0.16)	0.27 (0.15)
3.8 m/s	0.44 (0.07)	0.46 (0.18)	0.36 (0.05)	0.26 (0.10)	0.22 (0.17)	0.31 (0.14)

Note: Joint negative work data are in absolute values.

In the stance phase of walking, the middle age group had higher ankle joint plantar flexor moment angular impulse (*I*_*ankle*_) compared with the young age group (p = 0.002) across all walking speeds (Table 4). The middle age group also had a higher total support moment impulse (*I*_*total*_) compared with the young age group (p = 0.016) across all walking speeds. However, in the stance phase of running, there were no differences between young and middle age group for *I*_*joint*_ and *I*_*total*_ (Table 4).

**Table 4:** Stance phase joint extensor moment angular impulse (Nm·s/kg) and total support moment impulse (Nm s/kg) between young (n = 10) and middle age (n = 10) groups across walking and running speeds. Sample Mean (SD).

	Ankle^*^		Knee		Hip		Total^*^	
	Young	Middle	Young	Middle	Young	Middle	Young	Middle
***Walk***								
0.8 m/s	**0**.**53 (0**.**15)**	**0**.**53 (0**.**18)**	0.04 (0.05)	0.12 (0.08)	0.07 (0.04)	0.04 (0.03)	**0**.**65 (0**.**20)**	**0**.**67 (0**.**15)**
1.0 m/s	**0**.**43 (0**.**10)**	**0**.**52 (0**.**09)**	0.09 (0.12)	0.14 (0.11)	0.09 (0.05)	0.09 (0.02)	**0**.**61 (0**.**11)**	**0**.**75 (0**.**12)**
1.2 m/s	**0**.**36 (0**.**17)**	**0**.**54 (0**.**16)**	0.14 (0.14)	0.11 (0.08)	0.06 (0.04)	0.09 (0.04)	**0**.**56 (0**.**10)**	**0**.**74 (0**.**14)**
1.4 m/s	**0**.**39 (0**.**07)**	**0**.**43 (0**.**11)**	0.10 (0.05)	0.09 (0.08)	0.09 (0.02)	0.11 (0.06)	**0**.**59 (0**.**06)**	**0**.**63 (0**.**08)**
1.6 m/s	**0**.**30 (0**.**12)**	**0**.**54 (0**.**24)**	0.17 (0.16)	0.08 (0.03)	0.10 (0.05)	0.12 (0.05)	**0**.**56 (0**.**09)**	**0**.**69 (0**.**22)**
1.8 m/s	**0**.**30 (0**.**10)**	**0**.**46 (0**.**20)**	0.21 (0.22)	0.13 (0.08)	0.11 (0.03)	0.11 (0.03)	**0**.**62 (0**.**23)**	**0**.**70 (0**.**25)**
2.0 m/s	**0**.**29 (0**.**06)**	**0**.**38 (0**.**12)**	0.13 (0.06)	0.18 (0.18)	0.12 (0.04)	0.14 (0.02)	**0**.**53 (0**.**09)**	**0**.**69 (0**.**30)**
***Run***								
1.8 m/s	0.34 (0.08)	0.38 (0.06)	0.21 (0.06)	0.23 (0.06)	0.05 (0.01)	0.05 (0.04)	0.60 (0.06)	0.65 (0.09)
2.2 m/s	0.30 (0.12)	0.35 (0.05)	0.21 (0.04)	0.21 (0.06)	0.06 (0.02)	0.07 (0.03)	0.58 (0.14)	0.63 (0.08)
2.6 m/s	0.28 (0.12)	0.36 (0.04)	0.20 (0.08)	0.21 (0.08)	0.08 (0.06)	0.07 (0.03)	0.56 (0.11)	0.64 (0.07)
3.0 m/s	0.29 (0.12)	0.34 (0.05)	0.21 (0.05)	0.18 (0.07)	0.09 (0.04)	0.08 (0.03)	0.60 (0.11)	0.60 (0.09)
3.4 m/s	0.26 (0.13)	0.33 (0.06)	0.21 (0.05)	0.20 (0.13)	0.10 (0.06)	0.08 (0.04)	0.57 (0.11)	0.60 (0.10)
3.8 m/s	0.35 (0.06)	0.34 (0.05)	0.17 (0.04)	0.17 (0.05)	0.10 (0.03)	0.08 (0.04)	0.61 (0.06)	0.59 (0.06)

^*^Statistically significant difference between young and middle age groups across speeds are indicated in bold.

For joint angle comparison between two groups, there were no differences between young and middle age groups at either the ankle or hip joint (see Supplementary Table S4), however there were some significant differences at the knee joint. In the walking condition, middle age group had a higher knee flexion angle at ground contact (${\theta }_{knee}^{GCA}$) (p = 0.005) and toe off (${\theta }_{knee}^{TOA}$) (p < 0.001) across all walking speeds (Table 5). For ${\theta }_{knee}^{PEA}$ in walking, the young age group had a higher knee extension angle over the whole gait cycle across all walking speeds (p = 0.003) (Table 5). In running condition, the middle age group had a higher knee flexion angle at ground contact (${\theta }_{knee}^{GCA}$) compared with the young age group (p = 0.037) (Table 5). Similar to the walking condition the young age group had a higher ${\theta }_{knee}^{PEA}$ compared with the middle age group (p = 0.039) across all running speeds (Table 5).

**Table 5:** Knee joint angle (degree) between young (n = 10) and middle age (n = 10) groups across walking and running speeds. Sample Mean (SD).

	GCA*		TOA*		PFA		PEA*		ROM	
	Young	Middle	Young	Middle	Young	Middle	Young	Middle	Young	Middle
**Knee**										
*Walk*										
0.8 m/s	**−0**.**88 (4**.**40)**	**−7**.**95 (4**.**56)**	**−38**.**92 (6**.**86)**	**−44**.**07 (7**.**53)**	−65.04 (4.18)	−65.37 (3.87)	**1**.**22 (4**.**06)**	**−3**.**91 (3**.**26)**	66.26 (4.19)	61.46 (3.78)
1.0 m/s	**0**.**85 (4**.**66)**	**−7**.**07 (6**.**23)**	**−41**.**35 (8**.**42)**	**−48**.**09 (6**.**59)**	−67.74 (3.65)	−70.26 (6.95)	**2**.**82 (4**.**98)**	**−4**.**30 (5**.**74)**	70.55 (3.74)	65.96 (2.53)
1.2 m/s	**−0**.**18 (4**.**12)**	**−6**.**41 (5**.**14)**	**−36**.**21 (9**.**47)**	**−43**.**98 (11**.**51)**	−67.17 (3.91)	−70.04 (3.30)	**1**.**70 (3**.**72)**	**−2**.**82 (2**.**48)**	68.87 (3.14)	67.22 (2.91)
1.4 m/s	**−0**.**53 (4**.**60)**	**−7**.**16 (4**.**62)**	**−40**.**37 (3**.**95)**	**−40**.**95 (6**.**22)**	−67.32 (5.61)	−68.81 (4.29)	**1**.**43 (3**.**90)**	**−2**.**87 (2**.**38)**	68.75 (3.24)	65.94 (4.02)
1.6 m/s	**−3**.**75 (4**.**28)**	**−8**.**11 (4**.**47)**	**−35**.**02 (6**.**46)**	**−43**.**19 (11**.**32)**	−65.37 (5.59)	−70.65 (7.38)	**2**.**25 (4**.**28)**	**−3**.**41 (4**.**99)**	67.62 (3.26)	67.24 (4.43)
1.8 m/s	**−3**.**83 (4**.**23)**	**−6**.**92 (3**.**65)**	**−33**.**71 (7**.**46)**	**−41**.**62 (7**.**25)**	−64.83 (5.62)	−68.88 (5.46)	**1**.**75 (4**.**07)**	**−2**.**15 (2**.**78)**	66.58 (4.49)	66.74 (5.09)
2.0 m/s	**−6**.**07 (4**.**96)**	**−9**.**36 (6**.**55)**	**−33**.**99 (9**.**84)**	**−46**.**41 (5**.**39)**	−64.97 (5.43)	−71.31 (9.01)	**1**.**76 (4**.**19)**	**−3**.**90 (6**.**66)**	66.73 (4.12)	67.42 (5.21)
***Run***										
1.8 m/s	**−9**.**94 (5**.**05)**	**−18**.**03 (5**.**19)**	−18.35 (7.38)	−25.46 (8.80)	−77.44 (7.85)	−85.07 (11.81)	**−7**.**24 (5**.**56)**	**−14**.**65 (5**.**52)**	70.20 (8.12)	70.42 (11.93)
2.2 m/s	**−12**.**69 (4**.**09)**	**−17**.**23 (5**.**79)**	−17.58 (5.69)	−20.52 (6.58)	−87.58 (9.94)	−87.47 (12.20)	**−8**.**66 (3**.**63)**	**−13**.**97 (5**.**87)**	78.92 (10.74)	73.50 (12.27)
2.6 m/s	**−12**.**86 (4**.**61)**	**−17**.**77 (5**.**14)**	−16.96 (7.12)	−18.82 (6.48)	−95.67 (9.72)	−90.92 (11.99)	**−8**.**62 (4**.**61)**	**−12**.**82 (5**.**97)**	87.06 (11.86)	78.10 (11.97)
3.0 m/s	**−13**.**59 (6**.**23)**	**−19**.**07 (6**.**41)**	−15.29 (6.43)	−17.83 (5.93)	−104.06 (11.75)	−99.89 (11.79)	**−8**.**94 (4**.**99)**	**−14**.**09 (6**.**08)**	95.12 (12.11)	85.80 (10.86)
3.4 m/s	**−17**.**27 (6**.**07)**	**−21**.**88 (8**.**12)**	−18.02 (8.25)	−21.25 (9.56)	−111.86 (9.09)	−104.11 (13.13)	**−10**.**65 (4**.**89)**	**−15**.**12 (4**.**30)**	101.20 (8.91)	88.99 (12.17)
3.8 m/s	**−18**.**67 (6**.**52)**	**−21**.**91 (5**.**15)**	−15.82 (3.48)	−20.01 (4.98)	−120.17 (8.45)	−108.65 (9.20)	**−11**.**70 (4**.**27)**	**−17**.**07 (5**.**26)**	108.47 (7.09)	91.58 (9.96)

^*^Statistically significant difference between young and middle age groups across speeds, are indicated in bold. GCA: joint angle at ground contact; TOA: joint angle at toe off; PFA: joint peak flexion angle in whole gait cycle; PEA: joint peak extension angle in whole gait cycle; ROM: joint angle range of motion in whole gait cycle.

## Discussion

The goal of the current project was to identify ankle, knee and hip joint kinematic and kinetic characteristics between young and middle age healthy subjects while walking and running across speeds. Our results did not support the hypothesis that the middle age group would have higher joint stiffness. However, the hypothesis that the middle age group would generate more positive work at the proximal end of lower extremity in walking was supported.

For *K*_*ankle*_ in walking condition, there were no significant differences between the two groups. At speed 1.0–1.2 m/s, and 1.6–2.0 m/s, the middle age group was 37%, 32%, 60%, 29% and 37% higher than the young age group, and large effect size was found for each speed (d > 0.5), respectively (Table 1). In running condition, at speed 1.8 m/s, the middle age group was 38% higher (effect size d > 0.5) than the young age group. However, at speed 3.8 m/s, the middle age group was 17% lower than the young age group (Table 1). Similar trends were found for ${W}_{ankle}^{+}$ in walking stance phase. At speeds 1.2 m/s, and 1.6–2.0 m/s, ${W}_{ankle}^{+}$ for the middle age group was 44%, 34%, 28% and 28% higher compared with the young age group, and large effect size was found for each speed (d > 0.5), respectively (Table 2). These observations may indicate that when walking from medium to fast speeds, the middle age group would have a higher *K*_*ankle*_ value and this would likely be associated with the generation of a higher stance phase ${W}_{ankle}^{+}$ as well. Moreover, significantly higher *I*_*ankle*_ in the middle age group across all walking speeds would contribute to the higher percentage stance phase ${W}_{ankle}^{+}$ for the middle age group as well.

**Figure 1:**
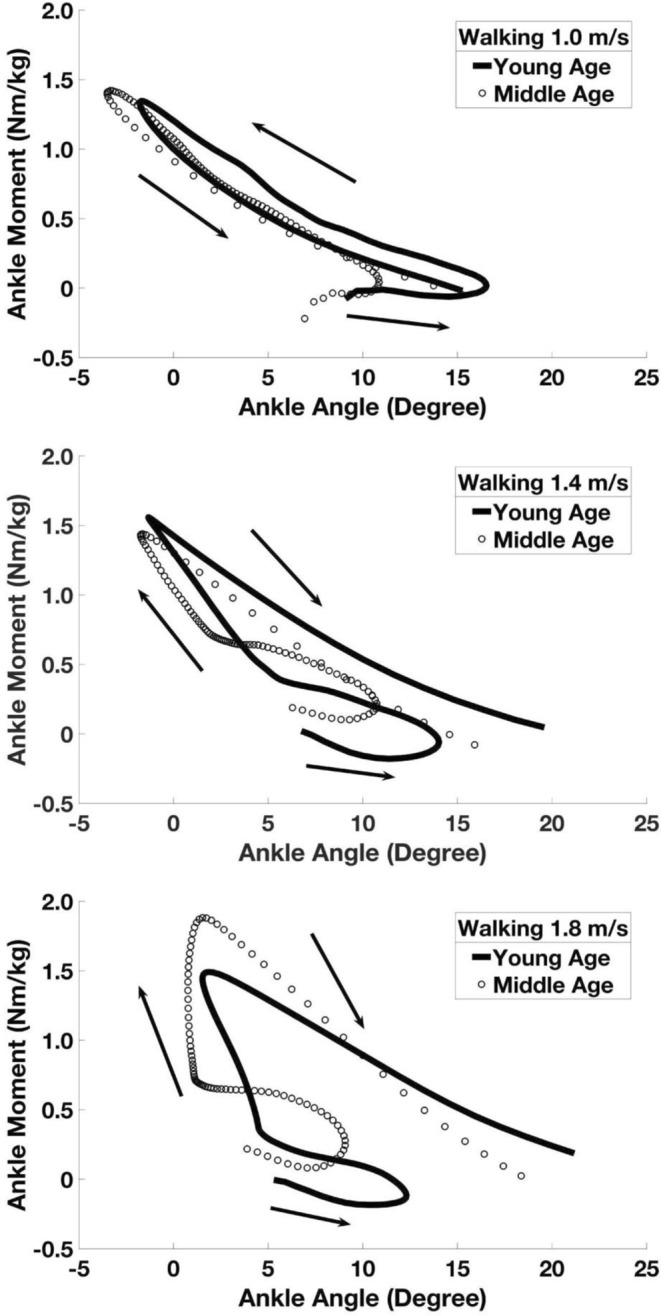
Group average ankle joint stance phase angle-moment curves between the young (n = 10) and middle age (n = 10) groups in three representative walking speeds.

**Figure 2:**
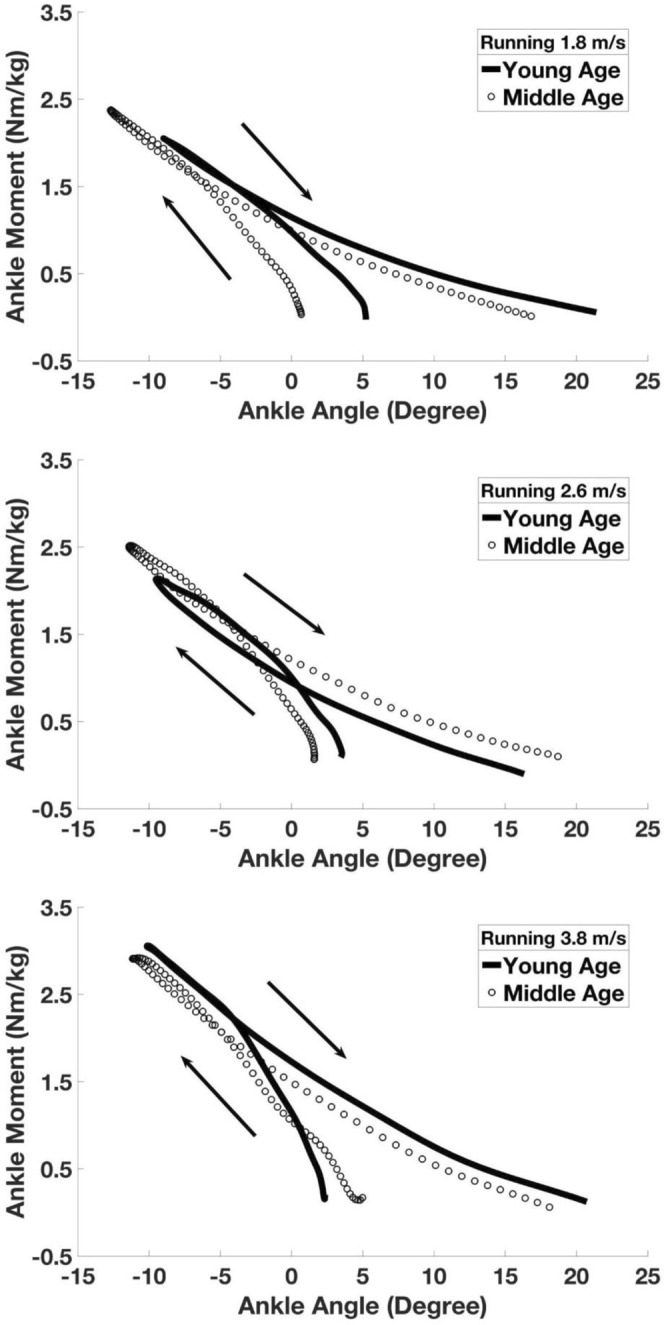
Group average ankle joint stance phase angle-moment curves between young (n = 10) and middle age (n = 10) groups in three representative running speeds.

In this study, we further examined ankle joint stance phase angle-moment relationship. We observed a clockwise hysteresis loop in both walking and running conditions. Both groups’ ankle angle-moment relationship agreed with previous studies in both walking and running conditions (Figs 1 and 2)^14–16^.

In the stance phase of walking, the middle age group had higher ${W}_{hip}^{+}$ (Table 2). This finding was similar to previous studies comparing an older age group and a young age group during walking^2,6^. This may be attributed to higher hip joint peak extensor moment and power during heel strike and loading response phase, and longer time for the middle age group subjects to extend the hip joint from a flexed positon to neutral position at midstance. This also indicates that the middle age group may take longer to achieve pelvis and trunk forward movement during early to midstance phase. Other studies have suggested that elderly people produce more positive work and extensor moment angular impulse at the hip joint to compensate for decreased ankle joint positive work and plantar flexor moment angular impulse to achieve similar gait performance^2,6,9^. In the present study, the middle age group produced higher *I*_*ankle*_ but not ${W}_{ankle}^{+}$. in walking. This may indicate an inefficient strategy for the middle age group. Specifically, ankle plantar flexor moment produced more *I*_*ankle*_, however this did not lead to higher ${W}_{ankle}^{+}$ generation among middle age individuals. This may result in the need of additional ${W}_{hip}^{+}$ to compensate for inefficient ${W}_{ankle}^{+}$ generation during walking. The middle age group may use an inefficient ankle strategy, along with a compensatory hip mechanical work strategy in walking. One possible reason may be related to the smaller age range in this study compared with previous studies comparing elderly and young groups, which reported that elderly rely more on a hip strategy^2^. The relatively narrow age range of participants involved in this study may contribute to the observation that the middle age group had a mixed strategy selection compared with young and elderly groups. Another reason might be that most of the middle age subjects in this study were generally fit (BMI: 23.0 ± 2.9 kg/m^2^). Relatively high fitness level may contribute to greater ankle plantar flexor moment response in stance phase among middle age subjects. The smaller age range and relatively high fitness level would both contribute to middle age subjects utilizing a mixed ankle and hip strategy to achieve the same locomotion task, compared with younger counterparts.

Comparison of joint kinematic patterns revealed that the middle age group had a higher knee joint flexion angle at ground contact and at toe off in walking, as well as less knee extension over the gait cycle in both walking and running compared with the young age group (Table 5). This observation coincides with the middle age group having a lower knee extensor moment and higher knee flexion velocity at ground contact and during the push-off period^17^, which would contribute to a greater knee flexion angle in the middle age group in both stance and swing phases of walking and running.

One limitation of the study is that gait symmetry was assumed. All subjects in both groups were healthy. The outcomes of this study may be only generalizable to the healthy young and middle age populations. Another limitation is that we controlled the walking and running speeds on the treadmill and thus some individual variations may have been restricted. Lastly, the middle age healthy volunteers from Oregon who responded to flyers to participate in this required study may not represent the overall middle age population of the USA.

Future studies should investigate lower extremity gait mechanics patterns among healthy young, middle and old age groups, and include comparisons with patient populations over a wider range of walking and running speeds. Moreover, further investigation of whole body center of mass kinetics among these populations during different locomotion tasks across speeds is needed.

## Conclusion

There were no difference of lower extremity joint stiffness between healthy young and middle age groups across different walking and running speeds. The middle age group had higher ankle plantar flexor moment angular impulse, total lower extremity support moment impulse and hip joint positive work in walking stance phase. The middle age group also exhibited higher knee flexion angle at ground contact. Based on these findings, it seems moderate aging does have effects on ankle and hip kinetic patterns across walking speeds, and on knee joint kinematic patterns in both walking and running.

## Methods

Ten healthy young subjects (5 female; 22.8 ± 5.3 years, 169.7 ± 11.2 cm, 67.2 ± 14.0 kg) and ten healthy middle age subjects (5 female; 50.7 ± 6.0 years, 173.4 ± 11.4 cm, 69.4 ± 14.9 kg) participated in the study. Detailed anthropometric data can be found in Supplementary Table S1. All subjects were without lower extremity musculoskeletal related injuries for the past 6 months before the test. Informed written consent was given by all participants, for the Institutional Review Board approved protocol. All methods were carried out in accordance with relevant guidelines and regulations. The research protocol was reviewed and approved by the office of Research Compliance Services at the University of Oregon (protocol #07302015.030).

Fifty-five retro-reflective markers were placed on the anatomical landmarks of the subjects, based on a previous study^18^. Firstly, participants were asked to walk on a force-instrumented treadmill (Bertec, Inc., Columbus, OH) at seven different speeds (0.8 to 2.0 m/s with 0.2 m/s intervals between each speed), and each walking speed condition lasted for 90 seconds. Then subjects were instructed to run at six different speeds, (1.8 to 3.8 m/s with 0.4 m/s intervals), and each running speed condition lasted for 75 seconds. Walking conditions were tested first, and there was a break between walking and running tests. Middle strides data were extracted (20 strides on average) from each condition for further analysis. We used an 8-camera motion capture system (Motion Analysis Corp., Santa Rosa, CA) to collect kinematic data, with a sampling rate at 120 Hz. A force-instrumented treadmill was used to collect ground reaction force (GRF) data, with a sampling rate at 1200 Hz. Marker position data were filtered with a low-pass fourth-order Butterworth filter at 6 Hz. Kinetic data were filtered with a low-pass fourth-order Butterworth filter at 50 Hz.

A standard inverse dynamics model was built in Visual 3D (C-Motion, Inc., Germantown, MD) to calculate ankle, knee and hip joint angles, moments and net joint powers. Joint stiffness $({K}_{joint})$ was estimated as sagittal plane change of joint moment $({\rm{\Delta }}{M}_{joint})$ divided by change of joint angular displacement ($({\rm{\Delta }}{\theta }_{joint})$) during the ground contact braking phase^16,19^, expressed as:

1 $${K}_{{joint}}=\frac{{\rm{\Delta }}{M}_{joint}}{{\rm{\Delta }}{\theta }_{joint}}$$

Lower extremity joint positive work (${W}_{joint}^{+}$) and negative work (${W}_{joint}^{-}$) were considered to be the sum of positive or negative net joint power integrated over time, respectively^20^. Stance phase joint extensor moment angular impulse (*I*_*joint*_) was calculated as the sum of all stance phase extensor (plantar-flexor for ankle) joint moment integrated over time^3,6^. Total lower extremity support moment impulse (${I}_{total}$) was calculated as the sum of ankle, knee and hip joint stance phase extensor moment angular impulse^3,6^, expressed as:

2 $${I}_{total}={I}_{ankle}+{I}_{knee}+{I}_{hip}$$

Joint level kinematic variables were calculated from the output results in Visual 3D. Joint ground contact angle $({\theta }_{joint}^{GCA})$ and toe-off angle $({\theta }_{joint}^{TOA})$ were chosen from the joint angle values in the first frame and the last frame of stance phase. Joint peak extension angle $({\theta }_{joint}^{PEA})$ and joint peak flexion angle $({\theta }_{joint}^{PFA})$ were chosen from the maximum extension and flexion joint angle in a whole gait cycle, respectively. Joint angle range of motion $({\theta }_{joint}^{ROM})$ was calculated as the difference between peak flexion angle and peak extension angle within the gait cycle. In this study, the sagittal plane neutral position of each joint was defined as the zero-degree reference angle, joint flexion as negative and joint extension as positive, compared with joint neutral position. The sagittal plane moment and net joint power calculation for each joint shared the same principle.

Ankle joint angle and moment values in stance phase were averaged within each group and plotted to further examine sagittal plane ankle dynamic loading in different walking and running speeds. We chose three different representative walking (1.0, 1.4, 1.8 m/s) and running (1.8, 2.6, 3.8 m/s) speeds, to show joint kinetic characteristics during slow, medium and relatively fast locomotion speed conditions.

All outcome variables were averaged from both left and right limbs, magnitude normalized to body mass and averaged across three different gait cycles. Joint stiffness (*K*_*joint*_), joint work (*W*_*joint*_), angular impulse (*I*_*joint*_) and all joint kinematic variables were compared in a 2-way mixed effects ANOVAs (group × speed) for each joint, within conditions, respectively, using SPSS (V22.0, IBM, Armonk, NY). The factor of group (young vs. middle age) was tested for between-subject effect and speed was tested for within subject effect in the statistical analysis. We set 0.05 as the initial alpha level. When we detected main effect or interaction effects, Bonferroni adjustments were used for post-hoc pairwise comparison. The post-hoc alpha level was adjusted based on the number of comparisons. Effect size comparison for *K*_*ankle*_, ${W}_{ankle}^{+}$ was conducted between young and middle age groups within each speed, respectively. Specifically, Cohen’s d effect size between the young and middle age group was calculated as the difference between the mean of the two groups divided by the mean of the standard deviation of the two groups in each condition. A Cohen’s d value between 0–0.2 is considered a small effect size, between 0.2–0.5 is a medium effect size, and a value higher than 0.5 is a large effect size.

## Supplementary information

Supplementary Data Tables

## Data Availability

Data may be made available upon request.
