# Supplementary material for: Comparison of lower extremity joint mechanics between healthy active young and middle age people in walking and running gait
Source: Sci Rep. 2019 Apr 3;9:5568. doi: 10.1038/s41598-019-41750-9 (PMC6447628; doi:10.1038/s41598-019-41750-9)
Supplement: Supplementary file 1 — Supplementary Data Tables [file 41598_2019_41750_MOESM1_ESM.docx]

**Comparison of lower extremity joint mechanics between healthy active young and middle age people in walking and running gait**

**Li Jin^1,2†^ & Michael E. Hahn^1,2*^**

^1^ Neuromechanics Laboratory

^2^ Bowerman Sports Science Clinic, Department of Human Physiology, University of Oregon, Eugene, OR, USA

^†^ Current appointment in Department of Physical Therapy & Rehabilitation Science, University of Iowa, Iowa City, IA, USA

**^*^**mhahn@uoregon.edu

|  | Mass (kg) | Height (cm) | Leg Length (cm) | Knee Width (cm) | Ankle Width (cm) |
| --- | --- | --- | --- | --- | --- |
| **Young** |  |  |  |  |  |
| S01 | 92.0 | 186.0 | 97.5 | 12.0 | 8.0 |
| S02 | 74.0 | 175.0 | 91.5 | 11.0 | 7.0 |
| S03 | 84.8 | 185.4 | 95.0 | 11.0 | 7.5 |
| S04 | 73.7 | 179.0 | 92.5 | 10.5 | 7.0 |
| S05 | 60.3 | 159.0 | 85.0 | 10.5 | 6.5 |
| S06 | 68.0 | 165.1 | 84.0 | 11.0 | 6.5 |
| S07 | 49.0 | 155.0 | 84.5 | 11.5 | 6.0 |
| S08 | 52.0 | 157.5 | 83.5 | 9.5 | 6.0 |
| S09 | 57.2 | 167.6 | 89.5 | 11.0 | 6.5 |
| S10 | 61.2 | 167.6 | 90.0 | 11.5 | 6.0 |
| **Middle** |  |  |  |  |  |
| S01 | 55.8 | 160.0 | 87.0 | 11.0 | 6.0 |
| S02 | 92.1 | 190.5 | 98.0 | 12.5 | 7.0 |
| S03 | 87.5 | 185.4 | 95.0 | 11.0 | 7.0 |
| S04 | 68.5 | 185.4 | 95.0 | 11.0 | 6.5 |
| S05 | 80.7 | 172.7 | 92.0 | 12.0 | 7.0 |
| S06 | 67.6 | 177.8 | 94.5 | 11.0 | 7.0 |
| S07 | 76.7 | 171.5 | 90.0 | 11.0 | 7.0 |
| S08 | 61.9 | 165.1 | 89.5 | 10.0 | 6.5 |
| S09 | 58.5 | 168.9 | 88.5 | 10.0 | 6.0 |
| S10 | 44.9 | 156.2 | 84.0 | 11.0 | 5.5 |

**Supplementary Table S1.** Anthropometric data (body mass, height, leg length, knee width, ankle width) of the young age (n = 10) and middle age (n = 10) group subjects.

|  | Ankle | |  | Knee | |  | Hip | |
| --- | --- | --- | --- | --- | --- | --- | --- | --- |
|  | Young | Middle |  | Young | Middle |  | Young | Middle |
| *Swing Phase Positive Work* |  |  |  |  |  |  |  |  |
| 0.8 m/s | <0.01 (0.00) | <0.01 (0.00) |  | 0.01 (0.01) | <0.01 (0.01) |  | 0.04 (0.02) | 0.04 (0.02) |
| 1.0 m/s | <0.01 (0.00) | <0.01 (0.00) |  | <0.01 (0.00) | 0.01 (0.00) |  | 0.06 (0.03) | 0.05 (0.01) |
| 1.2 m/s | <0.01 (0.00) | <0.01 (0.00) |  | <0.01 (0.00) | 0.01 (0.02) |  | 0.08 (0.03) | 0.07 (0.02) |
| 1.4 m/s | 0.01 (0.00) | 0.01 (0.00) |  | <0.01 (0.00) | 0.01 (0.00) |  | 0.08 (0.01) | 0.09 (0.02) |
| 1.6 m/s | 0.01 (0.00) | 0.01 (0.00) |  | 0.01 (0.01) | 0.01 (0.01) |  | 0.10 (0.03) | 0.10 (0.03) |
| 1.8 m/s | 0.01 (0.00) | 0.01 (0.00) |  | 0.01 (0.01) | 0.01 (0.01) |  | 0.13 (0.03) | 0.12 (0.03) |
| 2.0 m/s | 0.01 (0.00) | 0.01 (0.00) |  | 0.01 (0.01) | 0.01 (0.01) |  | 0.16 (0.04) | 0.16 (0.04) |
| *Swing Phase Negative Work* |  |  |  |  |  |  |  |  |
| 0.8 m/s | <0.01 (0.00) | <0.01 (0.00) |  | 0.10 (0.02) | 0.10 (0.02) |  | <0.01 (0.01) | <0.01 (0.00) |
| 1.0 m/s | <0.01 (0.00) | <0.01 (0.00) |  | 0.13 (0.02) | 0.13 (0.02) |  | <0.01 (0.00) | <0.01 (0.00) |
| 1.2 m/s | <0.01 (0.00) | <0.01 (0.00) |  | 0.16 (0.02) | 0.15 (0.02) |  | <0.01 (0.00) | <0.01 (0.00) |
| 1.4 m/s | <0.01 (0.00) | <0.01 (0.00) |  | 0.17 (0.01) | 0.16 (0.03) |  | <0.01 (0.00) | 0.01 (0.00) |
| 1.6 m/s | <0.01 (0.00) | <0.01 (0.00) |  | 0.18 (0.04) | 0.19 (0.03) |  | 0.01 (0.00) | 0.01 (0.01) |
| 1.8 m/s | <0.01 (0.00) | <0.01 (0.00) |  | 0.22 (0.03) | 0.22 (0.02) |  | 0.01 (0.01) | 0.02 (0.01) |
| 2.0 m/s | <0.01 (0.00) | <0.01 (0.00) |  | 0.26 (0.03) | 0.27 (0.03) |  | 0.02 (0.02) | 0.03 (0.01) |

**Supplementary Table S2.** Joint work (J/kg) between young (n = 10) and middle age (n = 10) groups in swing phase across walking speeds. Sample Mean (SD). Note: <0.01 indicates a negligible value; Joint negative work data were presented in absolute values.

|  | Ankle | |  | Knee | |  | Hip | |
| --- | --- | --- | --- | --- | --- | --- | --- | --- |
|  | Young | Middle |  | Young | Middle |  | Young | Middle |
| *Swing Phase Positive Work* |  |  |  |  |  |  |  |  |
| 1.8 m/s | 0.01 (0.00) | 0.01 (0.00) |  | 0.01 (0.01) | 0.01 (0.01) |  | 0.15 (0.02) | 0.18 (0.04) |
| 2.2 m/s | 0.01 (0.00) | 0.01 (0.00) |  | 0.01 (0.01) | 0.01 (0.01) |  | 0.22 (0.05) | 0.25 (0.05) |
| 2.6 m/s | 0.01 (0.00) | 0.01 (0.00) |  | 0.02 (0.01) | 0.01 (0.01) |  | 0.32 (0.06) | 0.33 (0.08) |
| 3.0 m/s | 0.01 (0.00) | 0.01 (0.00) |  | 0.02 (0.01) | 0.01 (0.01) |  | 0.44 (0.11) | 0.45 (0.07) |
| 3.4 m/s | 0.01 (0.00) | 0.02 (0.01) |  | 0.02 (0.02) | 0.01 (0.01) |  | 0.56 (0.12) | 0.53 (0.14) |
| 3.8 m/s | 0.02 (0.00) | 0.02 (0.00) |  | 0.03 (0.03) | 0.01 (0.01) |  | 0.67 (0.14) | 0.72 (0.16) |
| *Swing Phase Negative Work* |  |  |  |  |  |  |  |  |
| 1.8 m/s | <0.01 (0.00) | <0.01 (0.00) |  | 0.26 (0.03) | 0.27 (0.03) |  | 0.01 (0.01) | 0.01 (0.01) |
| 2.2 m/s | <0.01 (0.00) | <0.01 (0.00) |  | 0.35 (0.03) | 0.38 (0.05) |  | 0.02 (0.02) | 0.02 (0.01) |
| 2.6 m/s | <0.01 (0.00) | <0.01 (0.00) |  | 0.47 (0.06) | 0.48 (0.09) |  | 0.04 (0.02) | 0.03 (0.02) |
| 3.0 m/s | <0.01 (0.00) | 0.01 (0.00) |  | 0.58 (0.07) | 0.62 (0.10) |  | 0.05 (0.02) | 0.06 (0.04) |
| 3.4 m/s | 0.01 (0.00) | 0.01 (0.00) |  | 0.73 (0.08) | 0.73 (0.20) |  | 0.08 (0.03) | 0.09 (0.04) |
| 3.8 m/s | 0.01 (0.00) | 0.01 (0.00) |  | 0.88 (0.14) | 0.88 (0.18) |  | 0.08 (0.04) | 0.11 (0.05) |

**Supplementary Table S3.** Joint work (J/kg) between young (n = 10) and middle age (n = 10) groups in swing phase across running speeds. Sample Mean (SD). Note: <0.01 indicates a negligible value; Joint negative work data were presented in absolute values.

|  | GCA | |  | TOA | |  | PFA | |  | PEA | |  | ROM | |
| --- | --- | --- | --- | --- | --- | --- | --- | --- | --- | --- | --- | --- | --- | --- |
|  | Young | Middle |  | Young | Middle |  | Young | Middle |  | Young | Middle |  | Young | Middle |
| **Ankle** |  |  |  |  |  |  |  |  |  |  |  |  |  |  |
| *Walk* |  |  |  |  |  |  |  |  |  |  |  |  |  |  |
| 0.8 m/s | 11.19 (2.73) | 8.12 (4.37) |  | 11.59 (5.46) | 12.44 (4.83) |  | -1.60 (2.04) | -2.77 (3.50) |  | 20.85 (5.27) | 17.34 (4.59) |  | 22.45 (5.34) | 20.11 (3.77) |
| 1.0 m/s | 9.09 (2.50) | 6.93 (3.44) |  | 15.27 (4.45) | 15.21 (5.20) |  | -2.03 (3.50) | -3.82 (5.11) |  | 24.26 (5.81) | 20.40 (5.89) |  | 26.29 (5.24) | 24.21 (3.49) |
| 1.2 m/s | 8.56 (2.70) | 6.89 (2.47) |  | 14.13 (7.72) | 13.73 (6.72) |  | 1.62 (3.57) | 3.44 (2.33) |  | 24.22 (4.67) | 20.72 (4.88) |  | 25.84 (4.39) | 24.16 (4.21) |
| 1.4 m/s | 6.75 (2.78) | 6.29 (3.18) |  | 19.60 (4.96) | 15.91 (5.35) |  | -1.73 (4.23) | -2.37 (3.65) |  | 26.39 (5.10) | 22.37 (5.04) |  | 28.12 (4.52) | 24.75 (4.27) |
| 1.6 m/s | 6.31 (2.95) | 4.62 (2.92) |  | 19.69 (4.76) | 15.24 (4.46) |  | -0.69 (3.10) | -2.45 (3.88) |  | 26.90 (4.26) | 22.37 (5.40) |  | 27.60 (4.65) | 24.83 (4.17) |
| 1.8 m/s | 5.30 (2.73) | 3.89 (2.62) |  | 21.18 (5.96) | 18.37 (5.16) |  | 0.52 (2.22) | -1.93 (3.41) |  | 28.01 (5.27) | 23.34 (5.06) |  | 27.49 (5.12) | 25.27 (3.87) |
| 2.0 m/s | 2.98 (3.09) | 3.09 (3.02) |  | 21.42 (6.18) | 20.62 (4.26) |  | -0.43 (2.77) | -2.11 (7.10) |  | 27.09 (3.81) | 23.80 (5.33) |  | 27.52 (4.91) | 25.91 (3.47) |
| *Run* |  |  |  |  |  |  |  |  |  |  |  |  |  |  |
| 1.8 m/s | 5.22 (5.85) | 0.69 (3.73) |  | 21.40 (7.01) | 16.82 (4.95) |  | -9.42 (2.78) | -12.89 (3.51) |  | 26.63 (5.46) | 21.02 (5.26) |  | 36.05 (3.49) | 33.91 (4.35) |
| 2.2 m/s | 3.42 (5.22) | 1.51 (2.99) |  | 18.98 (10.84) | 18.23 (5.79) |  | -11.21 (3.65) | -12.49 (3.36) |  | 28.49 (6.24) | 24.95 (6.88) |  | 39.70 (3.60) | 37.44 (6.69) |
| 2.6 m/s | 3.45 (4.69) | 1.42 (3.39) |  | 21.30 (6.72) | 19.49 (6.14) |  | -10.28 (3.16) | -12.22 (3.58) |  | 30.30 (5.02) | 27.42 (7.64) |  | 40.58 (2.94) | 39.64 (7.25) |
| 3.0 m/s | 3.09 (5.03) | 2.81 (4.29) |  | 18.22 (6.47) | 16.86 (6.91) |  | -11.17 (2.57) | -11.94 (3.47) |  | 31.97 (4.66) | 28.01 (5.66) |  | 43.14 (4.76) | 39.95 (5.53) |
| 3.4 m/s | 2.33 (5.91) | 2.66 (5.69) |  | 16.00 (11.06) | 16.08 (7.34) |  | -11.60 (4.11) | -11.96 (4.54) |  | 31.60 (4.67) | 29.42 (5.51) |  | 43.20 (4.88) | 41.37 (5.40) |
| 3.8 m/s | 2.35 (4.32) | 4.98 (5.58) |  | 20.72 (5.06) | 18.10 (5.09) |  | -10.34 (3.84) | -11.26 (4.44) |  | 32.35 (4.20) | 31.39 (7.01) |  | 42.69 (4.80) | 42.65 (7.63) |
| **Hip** |  |  |  |  |  |  |  |  |  |  |  |  |  |  |
| *Walk* |  |  |  |  |  |  |  |  |  |  |  |  |  |  |
| 0.8 m/s | 0.78 (7.18) | 0.90 (9.95) |  | 21.47 (7.83) | 20.19 (10.30) |  | -5.91 (6.61) | -3.19 (10.43) |  | 29.70 (6.96) | 30.42 (10.39) |  | 35.61 (3.12) | 33.61 (3.95) |
| 1.0 m/s | -1.18 (7.50) | -1.56 (9.01) |  | 22.98 (7.35) | 23.32 (9.06) |  | -7.50 (5.57) | -6.14 (10.02) |  | 30.82 (5.50) | 32.99 (10.05) |  | 38.31 (2.71) | 39.13 (3.63) |
| 1.2 m/s | -3.29 (7.39) | -2.89 (9.20) |  | 27.92 (7.55) | 23.34 (13.27) |  | -8.06 (6.31) | -7.23 (10.42) |  | 33.86 (5.77) | 34.54 (10.26) |  | 41.93 (4.39) | 41.77 (3.38) |
| 1.4 m/s | -6.00 (7.41) | -5.20 (9.15) |  | 27.98 (5.70) | 28.16 (10.73) |  | -9.36 (6.29) | -8.05 (9.94) |  | 35.44 (4.94) | 35.47 (10.85) |  | 44.80 (3.89) | 43.52 (4.49) |
| 1.6 m/s | -8.67 (7.16) | -7.67 (8.66) |  | 31.44 (5.84) | 27.75 (15.82) |  | -10.50 (6.55) | -10.17 (9.67) |  | 37.43 (5.72) | 37.98 (10.20) |  | 47.93 (4.24) | 48.15 (5.44) |
| 1.8 m/s | -10.83 (7.17) | -8.53 (9.29) |  | 31.71 (7.48) | 27.59 (12.62) |  | -12.94 (6.45) | -10.94 (9.53) |  | 38.00 (4.95) | 37.51 (10.98) |  | 50.93 (4.51) | 48.45 (4.60) |
| 2.0 m/s | -13.94 (8.50) | -11.00 (9.95) |  | 31.19 (8.04) | 26.63 (12.10) |  | -15.49 (8.27) | -13.80 (10.79) |  | 38.10 (6.60) | 37.94 (9.73) |  | 53.59 (4.65) | 51.74 (6.27) |
| *Run* |  |  |  |  |  |  |  |  |  |  |  |  |  |  |
| 1.8 m/s | -1.01 (7.68) | -1.14 (10.17) |  | 25.45 (5.98) | 25.81 (10.28) |  | -8.10 (8.05) | -7.93 (11.18) |  | 26.22 (6.43) | 27.05 (10.53) |  | 34.32 (4.76) | 34.98 (4.81) |
| 2.2 m/s | -4.68 (6.94) | -3.94 (10.58) |  | 26.06 (7.01) | 29.19 (10.60) |  | -12.84 (8.10) | -11.07 (12.20) |  | 28.25 (6.44) | 29.85 (10.31) |  | 41.09 (6.15) | 40.92 (5.33) |
| 2.6 m/s | -5.77 (6.88) | -5.52 (10.72) |  | 27.00 (5.43) | 31.47 (12.16) |  | -16.80 (7.59) | -13.64 (11.31) |  | 29.88 (5.76) | 32.30 (11.74) |  | 46.68 (6.06) | 45.93 (5.56) |
| 3.0 m/s | -7.69 (7.30) | -7.36 (11.59) |  | 30.43 (5.51) | 33.11 (12.21) |  | -22.10 (8.26) | -18.53 (14.70) |  | 33.47 (5.44) | 34.65 (11.56) |  | 55.57 (6.83) | 53.18 (6.10) |
| 3.4 m/s | -10.17 (7.91) | -8.15 (12.92) |  | 29.63 (7.18) | 33.00 (17.03) |  | -26.67 (9.70) | -20.17 (15.87) |  | 34.22 (6.03) | 37.03 (13.09) |  | 60.88 (8.28) | 57.20 (8.68) |
| 3.8 m/s | -11.58 (6.68) | -12.00 (11.63) |  | 31.99 (7.77) | 33.74 (11.60) |  | -31.18 (8.72) | -26.21 (10.86) |  | 34.87 (6.56) | 35.55 (11.25) |  | 66.05 (6.66) | 61.75 (6.26) |

**Supplementary Table S4.** Ankle and hip joint angle (degree) between young (n = 10) and middle age (n = 10) groups across walking and running speeds. Sample Mean (SD). **GCA:** joint angle at ground contact; **TOA:** joint angle at toe off; **PFA:** joint peak flexion angle in whole gait cycle; **PEA:** joint peak extension angle in whole gait cycle; **ROM:** joint angle range of motion in whole gait cycle.
